# Study on Black Phosphorus Characteristics Using a Two-Step Thinning Method

**DOI:** 10.3390/ma15020615

**Published:** 2022-01-14

**Authors:** Qin Lu, Xiaoyang Li, Haifeng Chen, Yifan Jia, Tengfei Liu, Xiangtai Liu, Shaoqing Wang, Jiao Fu, Daming Chen, Jincheng Zhang, Yue Hao

**Affiliations:** 1 Key Laboratory of Advanced Semiconductor Devices and Materials, School of Electronic Engineering, Xi’an University of Posts & Telecommunications, Xi’an 710121, China; lxy974613873@sina.com (X.L.); chenhaifeng@xupt.edu.cn (H.C.); jiayifan@xupt.edu.cn (Y.J.); ltf001123@163.com (T.L.); liuxiangtai.1990@163.com (X.L.); wsqing1212@163.com (S.W.); 2 Departamento de Ingeniería Mecánica, Universidad de Santiago de Chile, Santiago 9160000, Chile; daming.chen@usach.cl; 3 Key Laboratory for Wide Band Gap Semiconductor Materials and Devices of Education, School of Microelectronics, Xidian University, Xi’an 710071, China; jchzhang@xidian.edu.cn (J.Z.); yhao@xidian.edu.cn (Y.H.)

**Keywords:** black phosphorus, mechanical exfoliation, ultraviolet radiation, annealing, field-effect transistor

## Abstract

A mild two-step method of black phosphorus (BP) flake thinning was demonstrated in this article. Slight ultraviolet–ozone (UVO) radiation followed by an argon plasma treatment was employed to oxidize mechanically exfoliated BP flakes and remove the surface remains of previous ozone treatment. The annealing process introduced aims to reduce impurities and defects. Low damage and efficient electronic devices were fabricated in terms of controlling the thickness of BP flakes through this method. These results lead to an important step toward the fabrication of high-performance devices based on two-dimensioned materials.

## 1. Introduction

Among 2D materials, black phosphorus (BP) exhibits a range of properties, such as a thickness-dependent bandgap [1,2], high carrier mobility [3], and extraordinary thermoelectric properties [4]. Various reports have shown the exceptional optical properties, optoelectronic properties, and biomedical performance of BP devices [5,6,7,8,9,10], which makes it a potential candidate for next-generation materials. However, the lack of a bottom-up approach for film fabrication limits its practical applications, and issues of the degradation of BP for applying a designed thickness to form high-quality surfaces have been investigated. Wet chemical and thermal treatment require not only controlling the thickness of BP flake but also maintaining its stability [11,12]. Conductive atomic force microscope anodic oxidation and electron beam sculpting were reported to accomplish nanopatterning and layer-by-layer thinning of BP [13,14]. Thermal oxidization [15,16] and plasma [17,18,19] treatment are established, commonly used thinning processes. Although using one of these thinning processes alone can thin down BP flakes, the residual oxidation products from oxidization and the non-uniformity of plasma dry thinning should be considered. Among these methods, UVO has displayed both low cost and easy control [20,21]. The oxide layer induced by UVO formed to form on the BP surface makes it more stable than that in ambient conditions but influences the electronic performance of devices.

To prevent this problem, we introduce in this work an effective, mild two-step method using black phosphorus (BP) flakes to form uniform and thin BP. BP flakes were first exposed to slight UVO radiation for a few minutes, and a mild argon plasma treatment followed. Steps were taken to reduce the impurities and defects from thinning through an annealing process in a N_2_ atmosphere. By controlling the UVO radiation, a stable thinning rate of exfoliated BP flakes was achieved. The residual oxidation products created from UVO can be easily removed by light plasma, and the defects induced during this process can be improved by the subsequent annealing process. Through the material surface roughness analysis, flattening surface was obtained through this two-step technique. Moreover, the performance of the BP field-effect transistor (FET) fabricated improved significantly.

## 2. Experimental Section

### Materials and Methods

**Figure 1:**
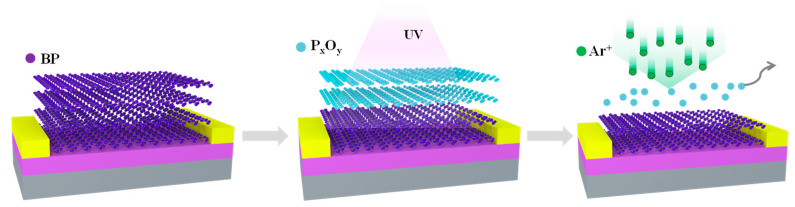
Schematic diagram of thinning process of the BP flakes. Slight UVO radiation was introduced to oxidize the BP flakes, and Ar plasma was used to remove the residue.

Figure 1 shows a schematic diagram of the thinning process of the BP flakes. BP flakes were obtained by mechanical exfoliation from a bulk BP crystal by Scotch tape, and following this, they were transferred directionally to source and drain electrodes (Ti/Au 5 nm/50 nm) which was on a 285 nm SiO_2_/Si substrate. To gain the target thickness, BP flakes were exposed to slight UVO through ultraviolet–ozone cleaning (SC-UV-I) equipment. Controlling the time of exposure to UVO light radiation (50 W) resulted in a thinning rate of about 2 nm/min. In this process, only the chemical reaction induced by the ozone oxidation can thin down the BP flakes without direct bombardment. Following this, light Ar atmosphere plasma was introduced using reactive ion etching (RIE) equipment to remove the residue on the top surface of the BP. After that, a 300 °C annealing process was used to repair and reduce the interface defects. The thickness and roughness of the flakes were measured by a Cypher S atomic force microscope (AFM) in tapping mode. Raman spectra characteristics were gathered using a LabRAM HR Raman spectrometer with an excitation wavelength of 514 nm. X-ray photoelectric spectroscopy (XPS) was used to analyze the binding energies of the BP flakes. The device’s electrical measurements were performed using an Agilent B1500A semiconductor parameter analyzer under vacuum conditions.

## 3. Results and Discussion

### 3.1. Material Characteristics

**Figure 2:**
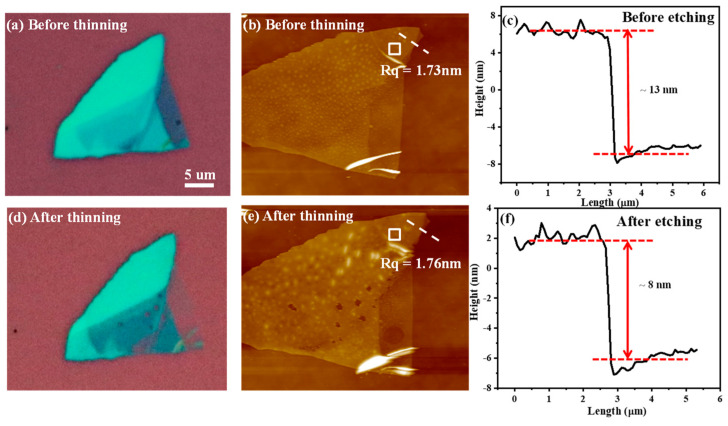
(**a**,**b**) Optical microscope and AFM image of BP flakes before thinning with a roughness of 1.73 nm at the square. (**c**) The thickness of the flake, which is −13 nm corresponding to the dotted line in (**b**,**d**,**e**), which are optical microscope and AFM images of BP flakes after thinning with a roughness of 1.76 nm at the square. (**f**) The thickness of the flake, which is −8 nm corresponding to the dotted line in (**e**).

Figure 2a,d show the optical microscopy images of the exfoliated BP flakes before and after light UV thinning and Ar treatment, respectively. The surface morphology and thickness were monitored by AFM, as shown in Figure 2b,c,e,f. Although the samples were vacuum-packed after experiments to avoid degradation, there were some small bubbles observed in the BP surface in optical microscopy images, shown in Figure 2b, which is due to airborne H_2_O and O_2_ during optical microscopy observation [22,23], and bubbles formed again more obviously during AFM measurements, shown in Figure 2e. Then, the BP sample was annealed in a N_2_ atmosphere for 1 min at 300 °C. The resultant sample, presented in Figure 2f, was measured to be ~8 nm thick by AFM, compared with the thickness of ~13 nm before thinning shown in Figure 2c. Meanwhile, the measured surface roughness of the thinned BP flakes is 1.76 nm, compared with 1.73 nm before thinning, indicating little change in the surface. Since the AFM characterization was performed after the Raman characterization and a high power of laser was used during the Raman test, this results in the broken point in the surface of the BP flakes in Figure 2e.

**Figure 3:**
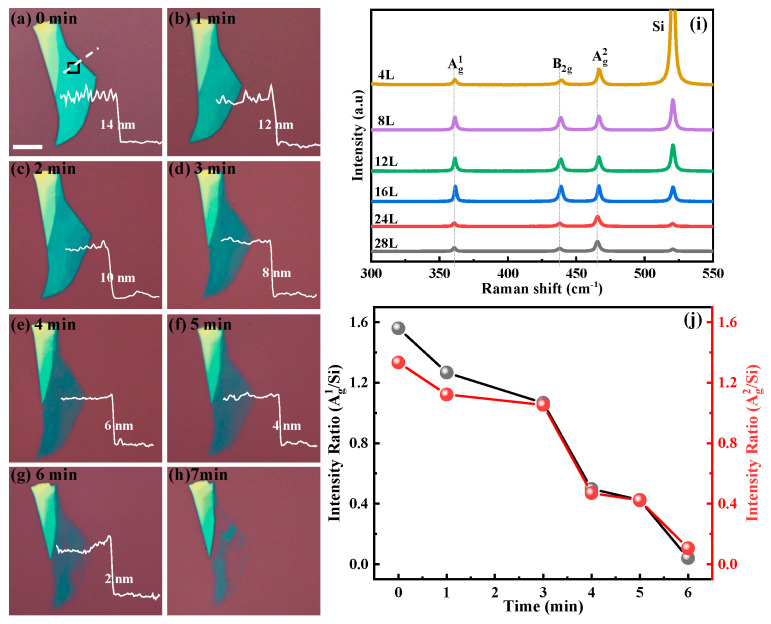
Optical images of mechanically exfoliated BP flakes treated with UV light radiation at different times and 300 °C annealing in N_2_ atmosphere (**a**–**h**). The length of the scale bar is 5 µm. Thinned thicknesses of BP flake as a function of the radiation time are shown as white lines. (**i**) Raman spectra of the BP flake being treated for various UVO light radiation corresponding to (**a**–**g**). (**j**) Intensity ratio of the A^1^_g_/Si and A^2^g/Si as functions of the UVO light radiation time.

The optical properties of the UVO and plasma-treated BP flakes were investigated using Raman spectroscopy. Figure 3a–h shows an optical microscopy image as a function of light UVO exposure time. With the increasing exposure time, the BP flakes decreased from 14 nm, and the thinning rate was about 2 nm/min, confirmed by AFM. Color and thickness of BP provide an effective method of monitoring the BP thickness during the thinning process. Figure 3i shows Raman spectra of the samples with varying light UVO exposure times, which corresponds to Figure 3a–g. In these spectra, three prominent peaks due to vibrations of the crystalline lattice matching the Raman shifts attributed to the A^1^_g_ (out-of-plane vibrations), B_2g_, and A^2^_g_ (in-plane vibrations) phonon modes of BP are located at 360.7, 437.4, and 465.6 cm^−1^, respectively. The spectra are normalized to the Raman peak of the A^2^_g_ peak at 465.6 cm^−1^ and indicate the retained crystalline structures of BP after UVO light and plasma treatment [24,25]. The peak intensities of all three phonon modes decreased, while the Raman peak of Si increased with increasing exposure time, which can also be seen through the ratio of the intensities of the A^1^_g_, A^2^_g_, and Si peak, respectively. In Figure 3j, both peak ratios of A^1^_g_/Si and A^2^_g_/Si used as a gauge decreased after longer exposure processes, indicating the thinning of the BP flakes [19,25].

**Figure 4:**
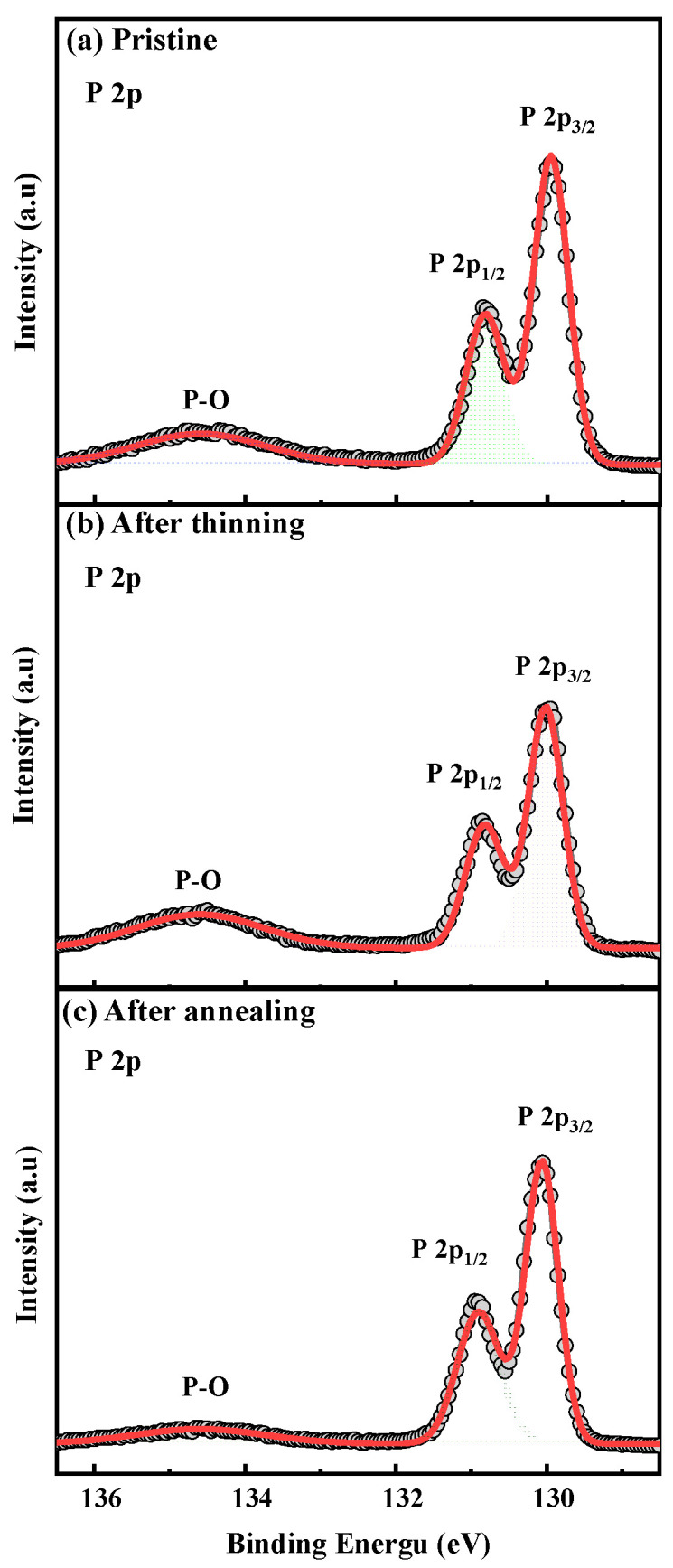
XPS spectra of P 2p. (**a**) Pristine; (**b**) after UV light and Ar plasma treatment; (**c**) after annealing.

Figure 4 displays the chemical bonding information on an element of the BP flake before and after treatment by X-ray photoemission spectroscopy (XPS). Survey XPS spectrum of BP flakes was shown in Figure S1. All the data are calibrated with the C 1s peak, which is recorded as constant binding energy of 284.6 ± 0.2 eV from the adventitious carbon. Before thinning treatment, the decomposition of the P element into two peaks was located to ~129.9 and 130.8 eV, assignable to 2p_3/2_ and 2p_1/2_ binding energy, respectively, shown in Figure 4a. The broadband located at ~134 eV is regarded as the contribution to BP oxidation during the treatment process, including P-O-P, O-P=O, and P_2_O_5_ [26]. As shown in Figure 4b, after treatment with UVO for 2 min and slight plasma, the intensity of the peaks at 2p_3/2_ and 2p_1/2_ decreased and the content of the P element changed from 44.63% in pristine to 39.51%, which shows that the thinning process worked. Additionally, thinning by this method induced an increase in the content of the oxygen element from 32.2% to 38.47%, and the intensity of peak at 134 eV increased. Moreover, after 300 °C annealing in a N_2_ atmosphere, the peak intensity corresponding to 134 eV decreased, shown in Figure 4c. The value of the calculated relative content of the P element is 41.5%, which reveals that the oxygen element introduced during the thinning process can be partly reduced by annealing. Despite oxidation occurring in BP flakes during thinning, the oxidation inversely contributes to the protection of BP [27].

### 3.2. Electronic Performance

**Figure 5:**
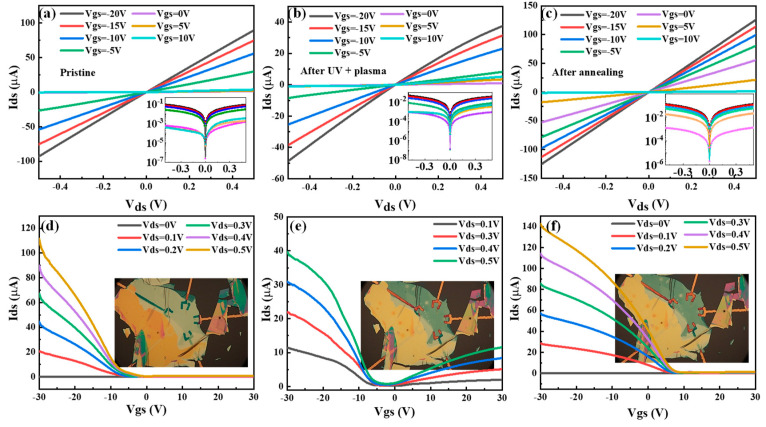
Current performance of BP flakes under different status which were tested at room temperature. I_ds_-V_ds_ characteristics of BP FET at pristine (**a**), after thinning by 5 min light UVO and slight Ar^+^ plasma treatment (**b**), and after 300 °C annealing in N_2_ atmosphere (**c**), with V_gs_ varying from −20 V to 10 V. Insets are log-scale plots with unit A on the y-axes. I_ds_-V_gs_ characteristics at pristine (**d**), after UVO and Ar^+^ plasma treatment (**e**), and after annealing (**f**), with V_ds_ varying from 0 V to 0.5 V. The insets are optical microscopy images of fabricated BP FETs under different treatments.

To investigate electrical device performance during the thinning process, a ~30 nm BP-based black-gate field-effect transistor (FET) on 300 nm SiO_2_/Si was fabricated and measured in vacuum conditions at room temperature. Figure 5a–c shows source–drain current (I_ds_)–source–drain bias (V_ds_) characteristics at pristine, after thinning, and after 300 °C annealing treatment, respectively. Insets are log-scale plots with unit ~A on the y-axes. After the thinning process, current modulations decrease and then increase after the annealing process. With variable black-gate (V_gs_) voltage from −20 V to 10 V, current modulations observed throughout the thinning process indicate ohmic contact performance, as confirmed by the linear I_ds_–V_ds_ curves. Figure 5d-f displays the transfer characteristics (I_ds_-V_gs_) of BP FET at pristine, after thinning, and after annealing, with V_ds_ changing from 0 V to 0.5 V, respectively. Insets show optical microscopy images of BP FETs during the thinning process corresponding to Figure 5a–c, respectively. The changed color of the BP flake indicates decreasing thickness. The device exhibits p-type dominant behaviors, which is in agreement with the previous reports [2,3]. After thinning under slight UVO radiation for 5 min and Ar+ plasma (step 1) and annealing at 300 °C in a N_2_ atmosphere for 1 min (step 2), the transfer curves of BP FET are dramatically changed, shown in Figure 5d-e. After the thinning process, the BP FET shows ambipolarity characteristics in the translated curve. It can be explained by the passivation protection of P_x_O_y_ introduced by the thinning process, which can be referred to previous reports on transistors with a weak n-type after BP passivation presentation [15,28]. The off-state current at high positive-gate voltage decreases, and the on/off current ratios (the ratio of the I_ds_ measured with and without the gate voltage when the V_ds_ is constant) increase significantly from 11 to 281 after steps 1 and 2. This result is due to the effective reduction of the channel thickness, which is consistent with the previous analysis of the AFM images. Further decreases in channel thickness will result in decreases in the interlayer resistance of the BP films and thus in increases in carrier conduction from the electrical contacts formed on the top surfaces of the BP films [29]. The mobility was estimated with the following formula, μ_eff = L/(W · C_g · V_ds ) · (I_ds/V_gs), to identify more about the change of BP FET performance under different processes. L and W are the length and width of the device channel, respectively, and Cg is the oxide capacitance per unit area (11.5 nF/cm^2^ for 300 nm SiO_2_) [30]. After step 1, the thickness of BP as-exfoliated decreased, and a strong interface state formed between BP and BP oxide layers that might trap electron conduction, which is consistent with the previous analysis of the XPS [31]. After step 2, thermal annealing reduced the trapping of carriers at interface defects induced by step 1 and was confirmed by the current curves in Figure 5f. The estimated mobility increases from 274 to 492 cm^2^ V^−^^1^·s^−^^1^ and is accompanied by the threshold voltage shift after thinning. These observations are attributed to p-type doping from atmospheric adsorbates, similar to effects observed with other 2D materials [22,32]. Due to the short time of thinning, the exfoliated material itself is not too thin to significantly enhance the mobility of BP FET. Further work needs to be done to investigate the performance of the device by this method.

### 3.3. Noise Performance

**Figure 6:**
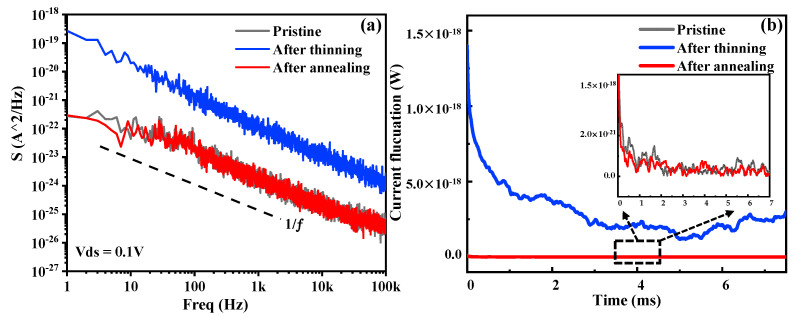
(**a**) Noise spectral density S(I^2^) for the BP device under different treatments at room temperature. The source-drain bias was 0.1 V. Note that the 1/f noise increases after step 1 and decreases after step 2. (**b**) Current fluctuations in the time domain at the same current values and temperature. Inset shows the current fluctuations between the pristine (black) and the sample after annealing (red).

To further validate the characteristics of the BP thinned by this method, we studied the noise performance of the sample. The same sample was put into a vacuum chamber at room temperature at every step of the experiment. Figure 6a shows the spectra density (SI^2^) under different treatments. Compared with the pristine, there is a slightly stronger noise density that occurred after BP thinning, which indicates impurities or defects on the induced BP. It is clear that the impurities or trap centers due to defects at the BP interface contribute significantly to noise density, which can be proven by XPS analysis of the changes above the P-O peak. Fortunately, it can be seen that the noise density obviously decreased after annealing treatment. Carriers from the impurity or defects we considered decreased during the annealing process, as well as the residues from device fabrication. On the other hand, the increase and decrease in carriers can also be proven by the current amplitude fluctuations, as shown in Figure 6b. The pristine sample indicates a small amount of fluctuation, but the sample after thinning shows a large one, which shows that the thinning process introduced defects and impurities even though it thinned the BP flakes [33,34,35,36]. After annealing, the current fluctuation due to carriers was effectively suppressed, as shown in the inset image, indicating a decrease in the defects or impurities on the BP. Consequently, the annealing process can improve the surface of BP, and this can be easily seen from the current fluctuations.

## 4. Conclusions

As a result, we explored a two-step method of thinning BP flakes with low damage. A slight UVO radiation was used to thin the flakes, and light plasma was induced to remove the residual oxidation of P on the top surface during thinning process. The flattened surface with low defect was obtained by annealing treatment. The on/off current and mobility of device based on BP indicate improvement after the thinning process. The two-step method provides an effective path to fabricating high-quality BP-based devices with a designed layer thickness.

## Data Availability

The data presented in this study are available on request from the corresponding author.

## Supplementary Materials

The following supporting information can be downloaded at https://www.mdpi.com/article/10.3390/ma15020615/s1, Figure S1: Survey XPS spectrum of BP flakes.
