# Supplementary material for: Study on Black Phosphorus Characteristics Using a Two-Step Thinning Method
Source: Materials (Basel). 2022 Jan 14;15(2):615. doi: 10.3390/ma15020615 (PMC8778055; doi:10.3390/ma15020615)
Supplement: Supplementary file 1 [file materials-15-00615-s001.zip › materials-1504551-supplementary.pdf]

materials

Article

Study on Black Phosphorus Characteristics Using a Two-Step Thinning Method

Qin Lu [1,*], Xiaoyang Li [1], Haifeng Chen [1], Yifan Jia [1], Tengfei Liu [1], Xiangtai Liu [1], Shaoqing Wang [1], Jiao Fu [1,*], Daming Chen [2], Jincheng Zhang [3] and Yue Hao [3]

[1] Key Laboratory of Advanced Semiconductor Devices and Materials, School of Electronic Engineering, Xi'an University of Posts & Telecommunications, Xi'an 710121, China; lxy974613873@sina.com (X.L.); chenhaifeng@xupt.edu.cn (H.C.); jiayifan@xupt.edu.cn (Y.J.); ltf001123@163.com (T.L.); liuxiangtai.1990@163.com (X.L.); wsqing1212@163.com (S.W.)

[2] Departamento de Ingeniería mecánica, Universidad de Santiago de Chile, Santiago 9160000, Chile; daming.chen@usach.cl

[3] Key Laboratory for Wide Band Gap Semiconductor Materials and Devices of Education, School of Microelectronics, Xidian University, Xi'an 710071, China; jchzhang@xidian.edu.cn (J.Z.); yhao@xidian.edu.cn (Y.H.)

* Correspondence: luqin@xupt.edu.cn (Q.L.); jiao_fu@foxmail.com (J.F.)

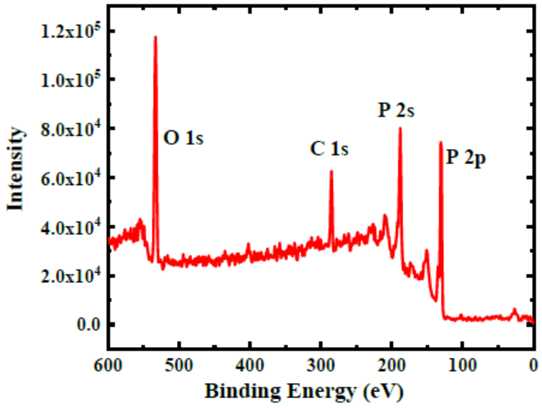

Figure S1. Survey XPS spectrum of BP flakes.

Figure S1 shows the survey spectrum of BP flakes including the peaks of C 1s, O 1s P 2s and P 2p.
